# Formulated Chinese Medicine Shaoyao Gancao Tang Reduces Tau Aggregation and Exerts Neuroprotection through Anti-Oxidation and Anti-Inflammation

**DOI:** 10.1155/2018/9595741

**Published:** 2018-10-28

**Authors:** I-Cheng Chen, Te-Hsien Lin, Yu-Hsuan Hsieh, Chih-Ying Chao, Yih-Ru Wu, Kuo-Hsuan Chang, Ming-Chung Lee, Guey-Jen Lee-Chen, Chiung-Mei Chen

**Affiliations:** ^1^Department of Neurology, Chang Gung Memorial Hospital, Chang Gung University College of Medicine, Taipei 10507, Taiwan; ^2^Department of Life Science, National Taiwan Normal University, Taipei 11677, Taiwan; ^3^Sun Ten Pharmaceutical Co. Ltd., New Taipei City 23143, Taiwan

## Abstract

Misfolded tau proteins induce accumulation of free radicals and promote neuroinflammation by activating microglia-releasing proinflammatory cytokines, leading to neuronal cell death. Traditional Chinese herbal medicines (CHMs) have been widely used in clinical practice to treat neurodegenerative diseases associated with oxidative stress and neuroinflammation. This study examined the neuroprotection effects of formulated CHMs Bai-Shao (made of *Paeonia lactiflora*), Gan-Cao (made of *Glycyrrhiza uralensis*), and Shaoyao Gancao Tang (SG-Tang, made of *P. lactiflora* and *G. uralensis* at 1 : 1 ratio) in cell model of tauopathy. Our results showed that SG-Tang displayed a greater antioxidative and antiaggregation effect than Bai-Shao and Gan-Cao and a stronger anti-inflammatory activity than Bai-Shao but similar to Gan-Cao. In inducible 293/SH-SY5Y cells expressing proaggregant human tau repeat domain (*Δ*K280 tau_RD_), SG-Tang reduced tau misfolding and reactive oxygen species (ROS) level in ΔK280 tau_RD_ 293 cells and promoted neurite outgrowth in ΔK280 tau_RD_ SH-SY5Y cells. Furthermore, SG-Tang displayed anti-inflammatory effects by reducing nitric oxide (NO) production in mouse BV-2 microglia and increased cell viability of ΔK280 tau_RD_-expressing SH-SY5Y cells inflamed by BV-2 conditioned medium. To uncover the neuroprotective mechanisms of SG-Tang, apoptosis protein array analysis of inflamed tau expressing SH-SY5Y cells was conducted and the suppression of proapoptotic proteins was confirmed. In conclusion, SG-Tang displays neuroprotection by exerting antioxidative and anti-inflammatory activities to suppress neuronal apoptosis in human tau cell models. The study results lay the base for future applications of SG-Tang on tau animal models to validate its effect of reducing tau misfolding and potential disease modification.

## 1. Introduction

Neurodegenerative diseases including Alzheimer's disease (AD) and tauopathy are characterized by the presence of hyperphosphorylated, insoluble, and filamentous tau protein, which leads to neuronal dysfunction and loss [1]. Tau is an ubiquitously distributed microtubule-associated protein that promotes and stabilizes microtubule assembly. Aside from helping microtubule assembly, tau also interacts with other cytoskeleton components to play a role in axonal transport [2]. Tau is encoded by *MAPT* (microtubule-associated protein tau) gene located on chromosome 17q21, containing 16 exons [3]. By alternative splicing, tau proteins exist as six different isoforms in human central nervous system (CNS). Exons 9–12 encode four C-terminal microtubule binding motifs which are imperfect copies of an 18-amino-acid tau repeat domain (tau_RD_). Different point mutations found in tau_RD_ reduced the ability of tau to promote microtubule assembly [4] and accelerated aggregation of tau into filaments [5]. In addition, a single amino acid deletion (*Δ*K280) was found in patients with frontotemporal dementia and AD [6–8]. ΔK280 is extremely fibrillogenic and frequently used to model tau aggregation [9–11].

Emerging evidence has shown protein aggregation as a trigger for inflammation and neurodegeneration [12]. Activated microglia are found in the postmortem brain tissues of human tauopathy, and microglial burden correlated with tau burden in most of the pathologically afflicted areas [13, 14]. Chronic activation of microglia may enhance the hyperphosphorylation of tau and the subsequent development of neurofibrillary tangles [15]. Activated microglia contribute to neurofibrillary pathology in AD through production of interleukin (IL)-1 and activation of neuronal p38-MAPK (mitogen-activated protein kinase 1) *in vitro* [16] and *in vivo* [17]. In transgenic mice that develop both tau and amyloid pathologies (3 × Tg-AD line), lipopolysaccharide- (LPS-) induced activation of glia exacerbates tau pathology [18]. Tau oligomers colocalize with astrocytes and microglia to induce inflammation, leading to neuronal damage and eventual cell death [19]. Being a critical component in pathogenesis, neuroinflammation provides an attractive therapeutic target in the treatment and prevention of AD and other tauopathy [20, 21].

Traditional Chinese herbal medicines (CHMs) have accumulated several lines of beneficial evidence in the treatment of AD [22–24]. However, treatment approaches addressing inflammatory processes in tauopathy have not been well investigated. Bai-Shao and Gan-Cao are formulated CHMs prepared from herbs *Paeonia lactiflora* (*P. lactiflora*) and *Glycyrrhiza uralensis* (*G. uralensis*), respectively. Total glucosides of paeony extracted from *P. lactiflora* may exert anti-inflammatory activities that contribute to its analgesic effect through modulating production of proinflammatory cytokines from macrophage-like synoviocytes [25]. In addition, ethanol extracts of *G. uralensis* possess inhibitory effects against NF-*κ*B-mediated inflammatory response and strong activation of the Nrf2-ARE-antioxidative stress signaling pathways [26]. In this study, Bai-Shao, Gan-Cao, and Shaoyao Gancao Tang (SG-Tang), a formulated CHM made of *P. lactiflora* and *G. uralensis* at 1 : 1 ratio, were tested in a tau aggregation model [27] to reveal underlying pathogenesis and develop therapeutic strategy targeting neuroinflammation in tauopathy.

## 2. Materials and Methods

### 2.1. Preparation of Formulated CHMs

Bai-Shao (Code: 5722), Gan-Cao (Code: 5536), and SG-Tang (Code: 0703H) were provided by Sun Ten Pharmaceutical Co. Ltd. (New Taipei City, Taiwan). To prepare the CHM stock solution, 5 g powder was dissolved in 10 ml ddH_2_O, vortexed to mix well, and then centrifuged at 4000 rpm for 10 min at room temperature. The supernatant was collected and used for further experiments.

### 2.2. HPLC Analysis

High-performance liquid chromatography (HPLC) was performed using a LaChrom Elite HPLC system (Hitachi, Tokyo, Japan) equipped with photodiode array detector. The chromatographic separation of Bai-Shao, Gan-Cao, and SG-Tang (500 mg/ml) was achieved using a Hypersil ODS (C18) column (250 × 4.6 mm, 5 *μ*m). The mobile phase consisted of 0.1% phosphoric acid in water (A) and acetonitrile (B). The linear gradient elution was used as follows: 10~50% B (0~40 min), 50~90% B (40~45 min), 90% B (45~55 min), 90~10% B (55~60 min), and 10% B (60~70 min). The flow rate was 0.8 ml/min. The column and autosampler were maintained at 30°C and 20°C, respectively. Reference compounds were paeoniflorin and ammonium glycyrrhizinate (Sigma-Aldrich, St. Louis, MO, USA) and absorbance was monitored at 230 nm and 250 nm, respectively. The scan range for photo diode array was 190~600 nm. 3-(4,5-Dimethylthiazol-2-yl)-2,5-diphenyltetrazolium bromide (MTT), 1,1-diphenyl-2-picrylhydrazyl (DPPH), LPS, and Congo red were purchased from Sigma-Aldrich. Interferon- (IFN-) *γ* was obtained from Santa Cruz.

### 2.3. Cell Culture

Two mouse cell lines, RAW 264.7 macrophage (BCRC 60001, Food Industry Research and Development Institute, Taiwan) and BV-2 microglia (kind gift from Dr. Han-Min Chen, Catholic Fu-Jen University, New Taipei City, Taiwan), were used in this study. The murine RAW 264.7 and microglial BV-2 cells were routinely maintained in DMEM supplemented with 10% FBS (Invitrogen, Waltham, MA, USA) at 37°C under 5% CO_2_ and 95% relative humidity.

Four human cell lines, HEK-293 cells (ATCC no. CRL-1573), SH-SY5Y neuronal cells (ATCC no. CRL-2266) and Tet-on ∆K280 tau_RD_-DsRed 293/SH-SY5Y cells [27] were used. HEK-293 cells were grown in DMEM with 10% FBS, and SH-SY5Y cells were maintained in DMEM-F12 with 10% FBS. In addition to the basal media for HEK-293 and SH-SY5Y, 5 *μ*g/ml blasticidin and 100 *μ*g/ml hygromycin (InvivoGen, San Diego, CA, USA) were applied for Tet-On ∆K280 tau_RD_-DsRed cells.

### 2.4. MTT Assay

To evaluate cell viability, 5 × 10^4^ HEK-293/SH-SY5Y cells were plated into 48-well dishes, grown for 20 h, and treated with tested Chinese medicine formulas (0.1~1000 *μ*g/ml Bai-Shao, Gan-Cao, or SG-Tang). After 1 day, 20 *μ*l of 5 mg/ml MTT was added onto each 48-well containing cells with 200 *μ*l of cultured medium at 37°C for 3 h. 200 *μ*l of lysis buffer (10% Triton X-100, 0.1 N HCl, 18% isopropanol) was then added onto 48-well and the absorbance at OD 570 nm was read by a microplate reader (FLx800 fluorescence microplate reader, Bio-Tek, Winooski, VT, USA). The half maximal inhibitory concentration (IC_50_) were calculated using the interpolation method.

### 2.5. DPPH Assay

The DPPH radical-scavenging activity was measured in a reaction mixture containing 0.1 ml of 0.2 mM DPPH radical solution and 0.1 ml of each tested formulas (100~1000 *μ*g/ml). The solution was rapidly mixed and incubated for 30 min at 25°C. The scavenging capacity was measured by monitoring the absorbance at 517 nm with a microplate reader (Multiskan GO, Thermo Scientific, Waltham, MA, USA). The half maximal effective concentrations (EC_50_) were calculated using the interpolation method.

### 2.6. Detection of Inflammatory Mediators

Murine RAW 264.7 macrophage cells were seeded in DMEM containing 1% FBS and pretreated with tested formulas (0.5~2 mg/ml) or celecoxib (50 *μ*M) for 8 h followed by LPS (1 *μ*g/ml) stimulation. The release of NO was evaluated by Griess assay according to the manufacturer's protocol (Sigma-Aldrich). The levels of tumor necrosis factor- (TNF-) *α*, IL-1*β*, and IL-6 were determined using a mouse enzyme-linked immunosorbent assay (ELISA) system (R&D Systems, Minneapolis, MN, USA) following the manufacturer's protocol. The optical density at 450 nm was detected using a microplate reader (ELISA Reader: SpectraMAX340PC; Molecular Devices, Sunnyvale, CA, USA). In addition, the immortalized murine microglial BV-2 cells, an alternative model system for primary microglia, were used. BV-2 cells were seeded in DMEM containing 1% FBS. Next day, cells were pretreated with SG-Tang for 8~24 h, stimulated with LPS (1 *μ*g/ml) for 20 h, and released of NO in the media determined.

### 2.7. ∆K280 tau_RD_-DsRed Fluorescence Assay

DsRed fluorescence was evaluated to reflect tau aggregation. On the first day, ∆K280 tau_RD_-DsRed 293 cells were seeded into the 96-well dish in a density of 0.8 × 10^4^ cells/well and one day after seeding, 5~20 *μ*M Congo red or 50~200 *μ*g/ml Bai-Shao, Gan-Cao, and SG-Tang were added. After 8 h of culture, doxycycline (1 *μ*g/ml; Sigma-Aldrich) was added to induce misfolded tau expression. On the fifth day, cells were stained with Hoechst 33342 (0.1 *μ*g/ml) for 30 min, and fluorescence intensities (543 nm excitation and 593 nm emission for DsRed; 377 nm excitation and 447 nm emission for Hoechst 33342) were measured using a high content analysis (HCA) system (ImageXpressMICRO, Molecular Devices). All images were analyzed by MetaXpress Image Acquisition and Analysis Software (Molecular Devices).

### 2.8. ROS Assay

Cellular ROS of the above Tet-On ∆K280 tau_RD_-DsRed 293 cells was measured by fluorogenic reagent (CellROX™ Deep Red, Molecular Probes, Eugene, OR, USA) with final concentration of 5 *μ*M and incubated at 37°C for 30 min. Then, cells were washed with PBS and analyzed by flow cytometer (Becton-Dickinson, Franklin Lakes, NJ, USA) with excitation/emission wavelengths at 640/665 nm. For each sample, 5 × 10^4^ cells are analyzed.

### 2.9. Neurite Outgrowth Analysis

3 × 10^4^ of ∆K280 tau_RD_-DsRed SH-SY5Y cells/well were seeded in a 24-well plate, and 10 *μ*M retinoic acid (Sigma-Aldrich) was added to initiate neuronal differentiation. On the second day, cells were treated with SG-Tang (200 *μ*g/ml) or Congo red (20 *μ*M) for 8 h before tau expression induction by adding doxycycline (1 *μ*g/ml). On day 9, cells were fixed in 4% paraformaldehyde in PBS for 15 min, permeabilized in 0.1% Triton X-100 in PBS for 10 min, and blocked in 3% bovine serum albumin (BSA) in PBS for 20 min. Primary TUBB3 antibody (1 : 1000 dilution in PBS with 1% BSA, 0.05% Tween 20, and 0.02% NaN_3_; Covance, Princeton, NJ, USA) was used to stain neuronal cells, followed by secondary goat anti-rabbit Alexa Fluor ®555 antibody (1 : 1000 dilution; Molecular probes) at room temperature. After nuclei staining by 4′-6-diamidino-2-phenylindole (DAPI), images of cells were taken via the HCA system and analyzed as described.

### 2.10. Cell Viability/Cytotoxicity Assays of Inflamed SH-SY5Y Cells

Previously, cell-free media obtained from LPS/IFN-*γ*-exposed microglia-like cells resulted in the highest toxicity on cell viability of SH-SY5Y cells [28]. To prepare conditioned medium (CM) with inflammatory factors, BV-2 cells were stimulated with a combination of LPS (1 *μ*g/ml) and IFN-*γ* (100 ng/ml) for 24 h. After morphology examination, the BV-2 CM were collected, pooled, and centrifuged to remove cell debris. The induced inflammation was confirmed by release of NO, TNF-*α*, IL-1*β*, and IL-6 in the media and increased Iba1 expression in the cell lysate.

For SH-SY5Y cell viability assay, DMEM-F12 was then mixed with two times volume of BV-2 CM (a final FBS concentration at 10%) and added to undifferentiated *Δ*K280 tau_RD_-DsRed SH-SY5Y cells for 2 days to induce inflammation. Cell viability was determined by MTT assay as described. For SH-SY5Y cytotoxicity assay, neuronal-differentiated ∆K280 tau_RD_-DsRed SH-SY5Y cells were treated with BV-2 CM for 5 days as described and media were collected. 100 *μ*l of supernatant from each sample was transferred to 96-well plate to examine the release of lactate dehydrogenase (LDH) by using LDH cytotoxicity assay kit (Cayman, Ann Arbor, MI, USA). The absorbance was read at 490 nm with a microplate reader (Multiskan GO, Thermo Scientific).

### 2.11. Human Apoptosis Antibody Array

Protein samples from *Δ*K280 tau_RD_-DsRed SH-SY5Y cells with different treatments (Dox uninduced/induced, CM unstimulated/stimulated, and SG-Tang unpretreated/pretreated) were prepared and incubated with apoptosis antibody array membranes (RayBiotech, Norcross, GA, USA). The relative levels of 43 apoptosis-related proteins in human cell lysates were measured with the array. The detected changes in protein levels were confirmed by Western blot or caspase 3 activity assay.

### 2.12. Western Blot Analysis

Cells were lysed in hypotonic buffer (20 mM HEPES pH 7.4, 1 mM MgCl_2_, 10 mM KCl, 1 mM DTT, 1 mM EDTA pH 8.0) containing the protease inhibitor mixture (Sigma-Aldrich). After sonication and sitting on ice for 20 min, the lysates were centrifuged at 14000 × *g* for 30 min at 4°C. Protein concentrations were determined using the Bio-Rad protein assay kit (Bio-Rad, Hercules, CA, USA), with albumin as standards. Total proteins (25 *μ*g) were electrophoresed on 10% or 12% SDS-polyacrylamide gel and transferred onto nitrocellulose membrane (Bio-Rad) by reverse electrophoresis. After being blocked, the membrane is stained with Iba1 (1 : 500; Wako, Osaka, Japan), Tau (1 : 200; Dako, Santa Clara, CA, USA), p-Tau Ser202 (1 : 200; Fremont, CA, USA), p-Tau Thr231 (1 : 500, Invitrogen), p-Tau Ser396 (1 : 500; Invitrogen), BID (1 : 1000; Cell Signaling, Danvers, MA, USA), BAD (1 : 500; Santa Cruz, Dallas, TX, USA), CYCS (1 : 500; Biovision, Milpitas, CA, USA), CASP8 (1 : 1000; Cell Signaling), DsRed (1 : 500; Santa Cruz), tubulin (1 : 1000; Sigma-Aldrich), or GAPDH (1 : 1000, MDBio) primary antibodies. The immune complexes are detected using horseradish peroxidase-conjugated goat anti-mouse (Jackson ImmunoResearch, West Grove, PA, USA) or goat anti-rabbit (Rockland, Pottstown, PA, USA) IgG antibody (1 : 10000 dilution) and ImmobilonTM Western Chemiluminescent HRP substrate (Millipore, Billerica, MA, USA).

### 2.13. Caspase 3 Activity Measurement

Cells were lysed in 1 × lysis buffer by repeated cycles of freezing and thawing. Caspase 3 activity was measured with the caspase 3 assay kit according to the manufacturer's instructions (Sigma-Aldrich).

### 2.14. Statistical Analysis

For each set of values, data are represented as mean ± SD of three independent experiments. Differences between groups were evaluated by two-tailed Student's *t*-test or ANOVA (one-way and two-way) with post hoc LSD test where appropriate. *p* values < 0.05 were considered significant.

## 3. Results

### 3.1. Formulated CHMs and Cytotoxicity

**Figure 1:**
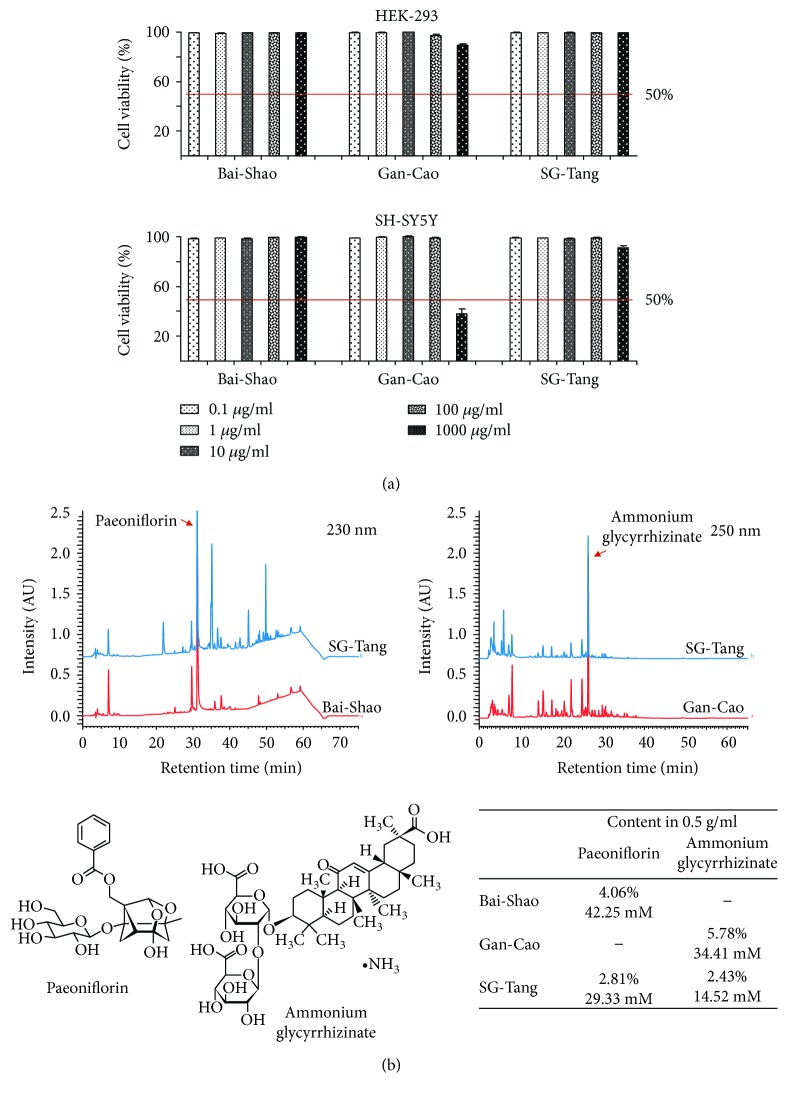
Cytotoxicity and chemical profiles of Bai-Shao, Gan-Cao, and SG-Tang. (a) MTT cell viability assay of HEK-293 and SH-SY5Y cells after treatment with Bai-Shao, Gan-Cao, and SG-Tang (0.1~1000 *μ*g/ml) for 24 h. To normalize, the relative viability of untreated cells was set as 100%. The red line represents 50% viability. (b) HPLC analysis of Bai-Shao, Gan-Cao, and SG-Tang. Chromatographic patterns (230 and 250 nm) show peaks compatible with paeoniflorin and ammonium glycyrrhizinate. Also shown below are chemical structures of paeoniflorin and ammonium glycyrrhizinate and the relative amounts (in % and mM) of these molecules in Bai-Shao, Gan-Cao, and SG-Tang (0.5 g/ml).

Three formulated CHMs, Bai-Shao, Gan-Cao, and SG-Tang were studied. To examine the cytotoxicity of these CHM formulas, MTT assay was performed on HEK-293 or SH-SY5Y cells after treatment with the tested formulas for 24 h. As shown in Figure 1(a), Bai-Shao, Gan-Cao, and SG-Tang exhibited very low cytotoxicity in HEK-293 and SH-SY5Y cells.

Next, the amounts of active constituents, paeoniflorin and ammonium glycyrrhizinate, in these CHM formulas were analyzed by full-spectrum analytic HPLC. As shown in Figure 1(b), chromatographic patterns showed peaks at 230 and 250 nm corresponding to the retention time compatible with paeoniflorin and ammonium glycyrrhizinate, respectively. The amounts of active constituents in these CHM formulas (0.5 g/ml) were 4.06% (42.25 mM) for paeoniflorin in Bai-Shao, 5.78% (34.41 mM) for ammonium glycyrrhizinate in Gan-Cao, and 2.81% (29.33 mM) for paeoniflorin and 2.43% (14.52 mM) for ammonium glycyrrhizinate in SG-Tang.

### 3.2. Radical-Scavenging Activity and Anti-Inflammatory Activity of the Tested Formulas

**Figure 2:**
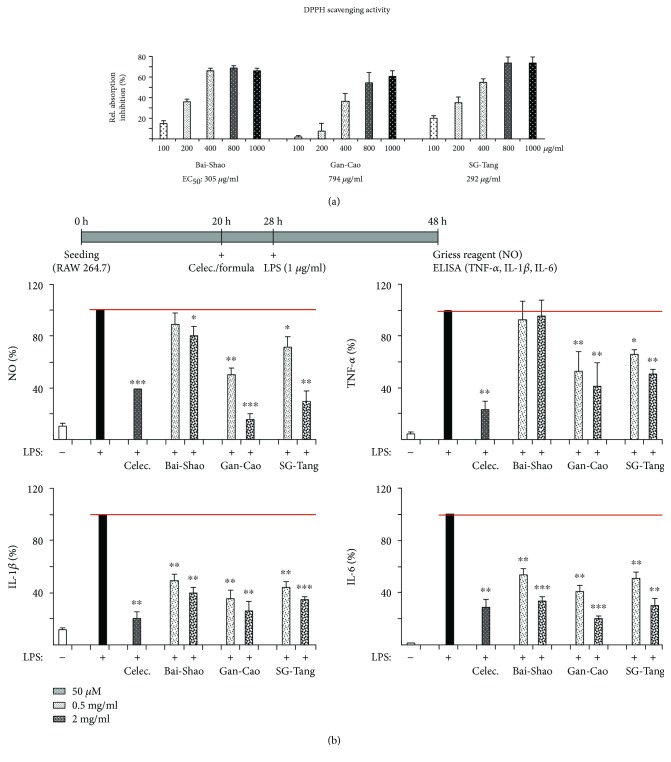
Antioxidative and anti-inflammatory activities of Bai-Shao, Gan-Cao, and SG-Tang. (a) DPPH radical-scavenging activities of the tested CHM formulas (100~1000 *μ*g/ml). The EC_50_ of each formula is shown under the columns. (b) Anti-inflammatory activities of the tested formulas on RAW 264.7 macrophages. Cells (10^6^) were pretreated with formulas (0.5~2 mg/ml) or compound celecoxib (Celec., 50 *μ*M) as a positive control for 8 h, and LPS (1 *μ*g/ml) was applied to induce inflammation. After 20 h, the levels of NO (assessed by Griess reagent), TNF-*α*, IL-1*β*, and IL-6 (assessed by ELISA) released into cultured media were determined (*n* = 3). For normalization, the relative NO, TNF-*α*, IL-1*β*, and IL-6 levels of LPS-treated cells were set as 100%. ^∗^ *p* < 0.05, ^∗∗^ *p* < 0.01, and ^∗∗∗^ *p* < 0.001, celecoxib/formulas treated vs. untreated cells.

To evaluate the radical-scavenging activity of these CHM formulas, DPPH scavenging assay was conducted. As shown in Figure 2(a), Bai-Shao, Gan-Cao, and SG-Tang displayed free radical-scavenging activities with EC_50_ at 305 *μ*g/ml, 794 *μ*g/ml, and 292 *μ*g/ml, respectively, indicating SG-Tang has a greater radical-scavenging activity than Bai-Shao or Gan-Cao. The anti-inflammatory responses of formulated CHMs were examined using RAW 264.7 cells, as LPS induced NO, TNF-*α*, and IL-6 production in murine macrophages [29, 30]. As shown in Figure 2(b), the exposure of RAW 264.7 cells to LPS resulted in a significant increase of NO, TNF-*α*, IL-1*β*, and IL-6 after 24 h of incubation (100% vs. 1~12%, *p* < 0.001). The elevations in NO, TNF-*α*, IL-1*β*, and IL-6 were reduced significantly in the presence of the nonsteroidal anti-inflammatory drug (NSAID) celecoxib (a selective cyclooxygenase (COX) inhibitor as a positive control) (NO: 39%, *p* < 0.001; TNF-*α*: 23%, *p* = 0.003; IL-1*β*: 20%, *p* = 0.001; IL-6: 29%, *p* = 0.002). Similar inhibitory phenomena were observed in the cells treated with Gan-Cao and SG-Tang (NO: 72~16%, *p* = 0.023~<0.001; TNF-*α*: 66~42%, *p* = 0.044~0.001; IL-1*β*: 44~26%, *p* = 0.004~<0.001; IL-6: 51~20%, *p* = 0.003~<0.001). Our results demonstrated that formulated CHMs Gan-Cao and SG-Tang possess anti-inflammatory effects by reducing production of inflammatory mediators.

### 3.3. Reduction of Tau Misfolding and Promotion of Neurite Outgrowth of the Tested Formulas

**Figure 3:**
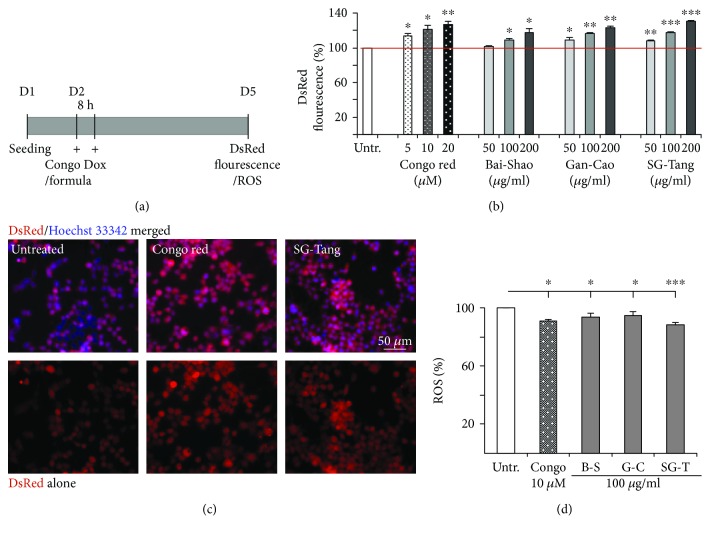
The effects of Bai-Shao, Gan-Cao, and SG-Tang on tau misfolding and ROS production in Tet-on *Δ*K280 tau_RD_-DsRed 293 cells. (a) Experiment flow chart. ΔK280 tau_RD_-DsRed 293 cells were pretreated with the tested formulas or Congo red (Congo, as a positive control) for 8 h before misfolded tau induction by doxycycline (Dox, 1 *μ*g/ml) for three days. (b) DsRed fluorescence analysis with Congo red (5~20 *μ*M) or the Chinese medicine formulas Bai-Shao, Gan-Cao, and SG-Tang (50~200 *μ*g/ml) treatment (*n* = 3). The relative DsRed fluorescence of untreated cells is normalized (100%). ^∗^ *p* < 0.05, ^∗∗^ *p* < 0.01, and ^∗∗∗^ *p* < 0.001, treated vs. untreated cells. (c) Representative microscopy images (upper row: merged DsRed and Hoechst 33342 signals; low row: DsRed signal alone) of ΔK280 tau_RD_-DsRed 293 cells untreated or treated with Congo red (20 *μ*M) or SG-Tang (200 *μ*g/ml). (d) ROS assay of ΔK280 tau_RD_-DsRed 293 cells untreated or treated with Congo red (10 *μ*M) or the tested formulas Bai-Shao (B-S), Gan-Cao (G-C), and SG-Tang (SG-T) (100 *μ*g/ml) (*n* = 3). The relative ROS of untreated cells was normalized as 100%. ^∗^ *p* < 0.05 and ^∗∗∗^ *p* < 0.001, treated vs. untreated cells.

Previously, we generated a proaggregant (*Δ*K280) tau_RD_ cell model targeting tau misfolding [27]. Inhibition of tau aggregation may improve DsRed misfolding, leading to increased fluorescence in tau_RD_-DsRed expressing cells. Utilizing the established Tet-on ΔK280 tau_RD_-DsRed 293 cells, Bai-Shao, Gan-Cao, and SG-Tang were tested for effects of reducing tau misfolding and antioxidation (Figure 3(a)). Fluorescent images of the cells were automatically recorded by a HCA system. As a positive control, Congo red (5~20 *μ*M) significantly increased the ∆K280 tau_RD_-DsRed fluorescence compared to no treatment (113~127% vs. 100%, *p* = 0.023~0.004). Significantly increased DsRed fluorescence was observed with Bai-Shao (109~117% for 100~200 *μ*g/ml treatment, *p* = 0.028~0.023), Gan-Cao (109~123% for 50~200 *μ*g/ml treatment, *p* = 0.017~0.003), and SG-Tang (108~130% for 50~200 *μ*g/ml treatment, *p* = 0.003~ < 0.001) compared to no treatment (Figure 3(b)). Representative fluorescent images of ∆K280 tau_RD_-DsRed cells untreated or treated with Congo red (20 *μ*M) or SG-Tang (200 *μ*g/ml) are shown in Figure 3(c). The results indicated that Bai-Shao, Gan-Cao, and SG-Tang reduced tau misfolding in our tauopathy 293 cell model and SG-Tang demonstrated a better antiaggregation function than Bai-Shao or Gan-Cao.

Misfolded tau may increase the production of reactive oxygen species (ROS) [31]. To examine whether these CHM formulas display antioxidative effects, ROS level was evaluated in Tet-On ∆K280 tau_RD_-DsRed 293 cells. As Figure 3(d) shows, pretreatment with Congo red (10 *μ*M, a positive control) or formulas (100 *μ*g/ml) significantly reversed the ROS level elevated by misfolded tau production compared to no treatment (88~95% vs. 100%, *p* = 0.045~<0.001). These data showed the anti-oxidative effects of Bai-Shao, Gan-Cao, and SG-Tang, and SG-Tang possesses a greater anti-oxidative effect than Bai-Shao or Gan-Cao.

**Figure 4:**
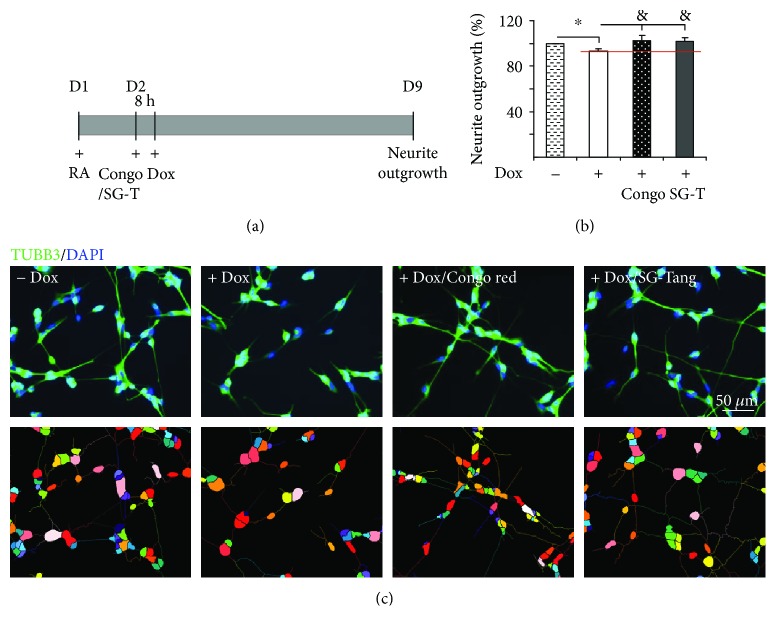
The effects of Bai-Shao, Gan-Cao and SG-Tang on neurite outgrowth in Tet-on *Δ*K280 tau_RD_-DsRed SH-SY5Y cells. (a) Experiment flow chart. ∆K280 tau_RD_-DsRed SH-SY5Y cells were seeded in 24-well (3 × 10^4^/well) plate with all *trans* retinoic acid (RA, 10 *μ*M). On day 2, cells were treated with Congo red (20 *μ*M) or SG-Tang (200 *μ*g/ml) for 8 h, induced tau_RD_-DsRed expression with doxycycline (Dox, 1 *μ*g/ml), and neurite outgrowth assayed on day 9. (b) Neurite outgrowth assay (*n* = 3) with Congo red or SG-Tang (SG-T) treatment. To normalize, the relative neurite outgrowth of untreated cells is set as 100%. ^∗^ *p* < 0.05, induced vs. un-induced cells; ^&^*p* < 0.05, treated vs. untreated cells. (c) Representative microscopy images of neuronally differentiated ΔK280 tau_RD_-DsRed SH-SY5Y cells uninduced (− Dox), untreated (+ Dox), and after treatment with Congo red (+ Dox/Congo red) or SG-Tang (+ Dox/SG-Tang). Neurites were stained with TUBB3 (neuronal class III *β*-tubulin, green) antibody. Nuclei were detected using (DAPI, blue). Upper row, merged TUBB3 and DAPI signals; lower row, images of the neurites and the body of individual cells being outlined by the same color for outgrowth quantification.

Since our study showed that SG-Tang has greater effects in free radical scavenging, antioxidation, and antiaggregation than Bai-Shao or Gan-Cao, we focused on SG-Tang treatment in subsequent experiments. The neuroprotective potential of SG-Tang was examined (Figure 4(a)). As Figure 4(b) shows, misfolded tau induction significantly reduced the length of neurites as compared to the absence of induction (93% vs. 100%, *p* = 0.030) and 20 *μ*M Congo red (positive control) or 200 *μ*g/ml SG-Tang pretreatment ameliorated this negative effect (103% vs. 93%, *p* = 0.041 for Congo red; 101% vs. 93%, *p* = 0.039 for SG-Tang). Representative neurite outgrowth images uninduced (− Dox), untreated (+ Dox), and after treatment with Congo red and SG-Tang are shown in Figure 4(c). Thus, SG-Tang exerts neuroprotective effect by rescuing the reduction of neurite outgrowth induced by tau misfolding.

### 3.4. Anti-Inflammatory Effects of SG-Tang in LPS-Stimulated BV-2 Microglia

**Figure 5:**
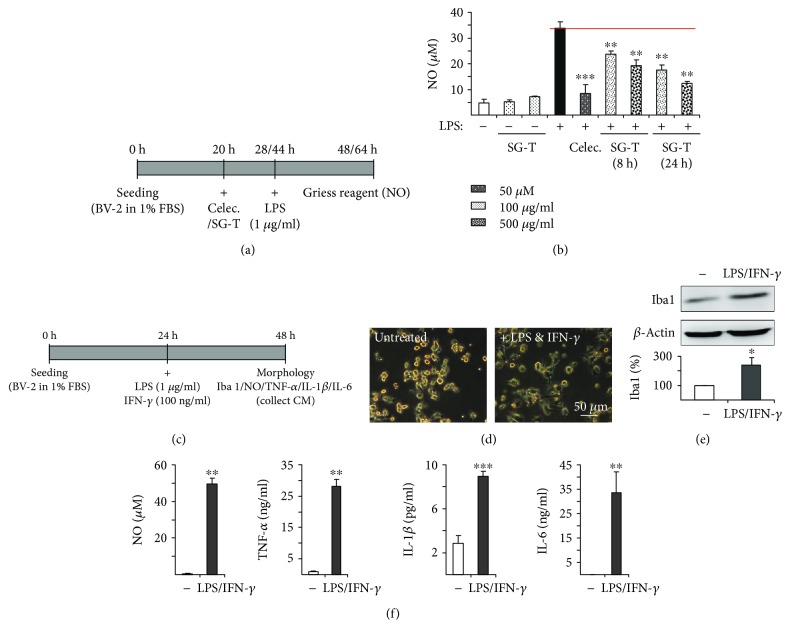
Anti-inflammatory effects of SG-Tang and BV-2 conditioned medium preparation. (a) Experiment flow chart for LPS stimulation. BV-2 cells were seeded in 1% fetal bovine serum (FBS) and pretreated with 50 *μ*M celecoxib (Celec.) for 8 h or 100~500 *μ*g/ml SG-Tang (SG-T) 8~24 h followed by 1 *μ*g/ml LPS stimulation 20 h. NO level was evaluated with Griess reagent. (b) Anti-inflammatory effect of celecoxib (Celec.) and SG-Tang (SG-T) on BV-2 cells (*n* = 3). ^∗∗^ *p* < 0.01 and ^∗∗∗^ *p* < 0.001, treated vs. untreated cells. (c) Experiment flow chart for LPS/IFN-*γ* stimulation. For preparation of BV-2 conditioned medium (CM), BV-2 cells were seeded in Dulbecco's modified Eagle's medium (DMEM) with 1% FBS medium. Next day, cells were stimulated with a combination of LPS (1 *μ*g/ml) and IFN-*γ* (100 ng/ml). After 24 h stimulation, the BV-2 CM was collected and examined for inflammation by morphology, Iba1 Western blotting and NO/TNF-*α*/IL-1*β*/IL-6 determination. (d) Morphology of BV-2 cells. (e) Western blot analysis of Iba1 expression in inflamed BV-2 cells (*n* = 3). To normalize, Iba1 expression level in uninflamed cells was set as 100%. ^∗^ *p* < 0.05, stimulated vs. unstimulated cells. (f) Secretion of NO, TNF-*α*, IL-1*β*, and IL-6 in BV-2 CM. ^∗∗^ *p* < 0.01 and ^∗∗∗^ *p* < 0.001, stimulated vs. unstimulated cells.

In the brain, activated microglia release proinflammatory mediators such as NO and cytokines as a response to inflammation [32]. Thus, the anti-inflammatory effects of SG-Tang were determined using LPS-stimulated BV-2 microglia (Figure 5(a)).  Figure 5(b) demonstrates that NO production of BV-2 cells significantly increased by LPS stimulation (33.9 *μ*M vs. 4.8 *μ*M, *p* < 0.001) and pretreatment of 100~500 *μ*g/ml SG-Tang for 8~24 h significantly reduced NO production (100 *μ*g/ml for 8 h: 23.7 *μ*M, *p* = 0.008; 500 *μ*g/ml for 8 h: 19.4 *μ*M, *p* = 0.002; 100 *μ*g/ml for 24 h: 17.5 *μ*M, *p* = 0.001; 500 *μ*g/ml for 24 h: 12.2 *μ*M, *p* = 0.002). The results indicate that SG-Tang displayed anti-inflammatory effects by reducing NO production in microglia. We then applied LPS and IFN-*γ* to BV-2 cells for 24 h for conditioned medium (CM) collection (Figure 5(b)). The resting BV-2 microglia showed a ramified morphology but more extended processes with elongated morphology were observed after LPS/IFN-*γ* treatment for 24 h (Figure 5(d)). As shown in Figures 5(e) and 5(f), elevated Iba1 (induction of brown adipocytes 1, a microglial marker) expression in inflamed BV-2 cells (100% vs. 240%, *p* = 0.042) and increased release of NO, TNF-*α*, IL-1*β*, and IL-6 in BV-2 CM (NO: 0.5 *μ*M vs. 49.6 *μ*M, *p* = 0.001; TNF-*α*: 0.9 ng/ml vs. 28.1 ng/ml, *p* = 0.002; IL-1*β*: 2.9 pg/ml vs. 8.9 pg/ml, *p* < 0.001; IL-6: 0 ng/ml vs. 33.6 ng/ml, *p* = 0.021) were confirmed. The collected CM was then used to provide inflammatory mediators to ∆K280 tau_RD_-DsRed SH-SY5Y cells.

### 3.5. Effects of SG-Tang on BV-2 Conditioned Medium-Inflamed ∆K280 tau_RD_-DsRed SH-SY5Y Cells

**Figure 6:**
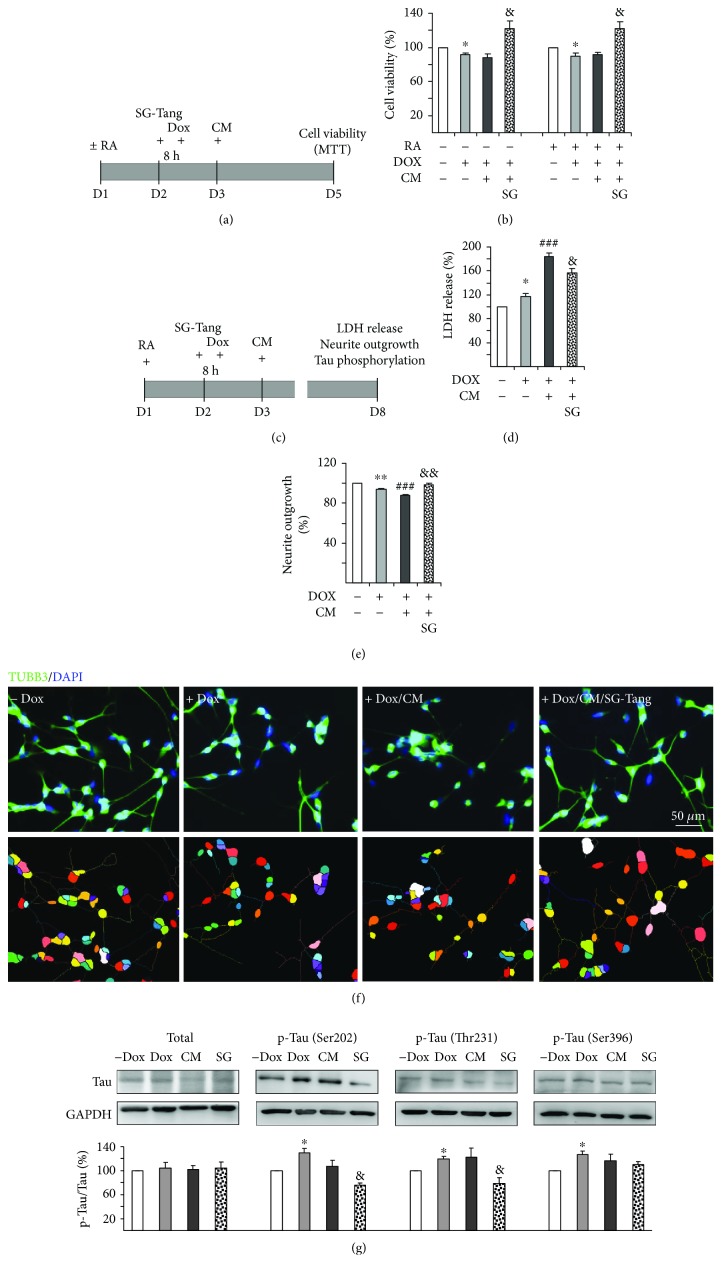
Neuroprotection of SG-Tang on ∆K280 tau_RD_-DsRed SH-SY5Y cells from BV-2 conditioned medium-induced cell death. (a) Experiment flow chart for cell viability assay. ∆K280 tau_RD_-DsRed SH-SY5Y cells were plated in media with/without retinoic acid (± RA, 10 *μ*M) on day 1 and pretreated with SG-Tang the next day for 8 h, followed by doxycycline addition (Dox, 1 *μ*g/ml) to induce misfolded tau expression. On day 3, DMEM-F12 media was mixed with BV-2 CM and cell viability was assessed by MTT assay on day 5. (b) Cell viability assay (^∗^ *p* < 0.05, **−** Dox vs. + Dox; ^&^*p* < 0.05, + Dox/CM vs. + Dox/CM/SG-Tang-treated cells) (*n* = 3). (c) Experiment flow chart for LDH release, neurite outgrowth, and tau phosphorylation assays. RA (10 *μ*M, present in cultures throughout) differentiated ∆K280 tau_RD_-DsRed SH-SY5Y cells were pretreated with SG-Tang (200 *μ*g/ml) on day 2 for 8 h, followed by inducing ∆K280 tau_RD_-DsRed expression (+ Dox, 1 *μ*g/ml). On day 3, DMEM-F12 was mixed with BV-2 CM and added to the cells. After five days, media were collected for LDH release examination. In addition, cells were examined for neurite outgrowth and tau phosphorylation. (d) LDH assay (^∗^*p* < 0.05, **−** Dox vs. + Dox; ^###^*p* < 0.001, + Dox vs. +Dox/CM; ^&^*p* < 0.05, + Dox/CM vs. + Dox/CM/SG-Tang treated cells) (*n* = 3). (e) Neurite outgrowth assay (*n* = 3). To normalize, the relative neurite outgrowth of uninduced cells is set as 100%. ^∗∗^ *p* < 0.01, **−** Dox vs. + Dox; ^###^*p* < 0.001, + Dox vs. + Dox/CM; ^&&^*p* < 0.01, + Dox/CM vs. + Dox/CM/SG-Tang-treated cells. (f) Representative microscopy images of differentiated *Δ*K280 tau_RD_-DsRed SH-SY5Y cells uninduced (− Dox), induced (+ Dox), inflamed (+ Dox/CM), or treated with SG-Tang (+ Dox/CM/SG-Tang). Neurites were stained with TUBB3 (green) antibody. Nuclei were detected using DAPI (blue). Upper row, merged TUBB3 and DAPI signals; lower row, images of the neurites and the body of a cell being outlined by the same color for outgrowth quantification. (g) Western blot analysis of total and phosphorylated (Ser202, Thr231, and Ser396) tau (normalized to GAPDH internal control, *n* = 3). ^∗^ *p* < 0.05, **−** Dox vs. + Dox; ^&^*p* < 0.05, + Dox/CM vs. + Dox/CM/SG-Tang-treated cells.

Undifferentiated (without retinoic acid, − RA) or differentiated (with retinoic acid, + RA) ∆K280 tau_RD_-DsRed SH-SY5Y cells were pretreated with SG-Tang (200 *μ*g/ml) for 8 h before misfolded tau induction and then BV-2 CM was added to provoke inflammatory damage on SH-SY5Y cells for two days (Figure 6(a)).  Figure 6(b) shows that misfolded tau induction reduced the viability of ∆K280 tau_RD_-DsRed SH-SY5Y cells (− RA: 91% vs. 100%, *p* = 0.012; + RA: 90% vs. 100%, *p* = 0.035) and application of SG-Tang rescued the decreased cell viability caused by misfolded tau induction and BV-2 CM addition (− RA: 122% vs. 88%, *p* = 0.015; + RA: 122% vs. 91%, *p* = 0.014). Thus, the reduced viability of inflamed ∆K280 tau_RD_-DsRed SH-SY5Y cells was not influenced by retinoic acid.

Differentiated SH-SY5Y cells expressing ∆K280 tau_RD_-DsRed were further evaluated on day 8 for LDH release, neurite outgrowth, and tau phosphorylation (Figure 6(c)). Both addition of Dox (118% vs. 100%, *p* = 0.019) and BV-2 CM (184% vs. 118%, *p* < 0.001) increased the LDH release of ∆K280 tau_RD_-DsRed SH-SY5Y cells and application of SG-Tang attenuated the LDH release (156% vs. 184%, *p* = 0.010) (Figure 6(d)). Misfolded tau induction significantly reduced the length of neurites compared to the uninduced cells (94% vs. 100%, *p* = 0.005), and addition of BV-2 CM aggravated this condition (88% vs. 94%, *p* < 0.001). Pretreatment of SG-Tang resulted in significant increase of neurite outgrowth (98% vs. 88%, *p* = 0.004) (Figure 6(e)). Representative images of neurite outgrowth of the above cells are shown in Figure 6(f).

The abnormal hyperphosphorylation of tau plays a role in the molecular pathogenesis of AD and other tauopathies. Therefore, the amount of phosphorylated tau was examined and Western blot showed that misfolded tau induction increased tau phosphorylation at residue Ser202, Thr231, and Ser396 compared to uninduced cells (Ser202: 130% vs. 100%, *p* = 0.020; Thr231: 119% vs. 100%, *p* = 0.016; Ser396: 127% vs. 100%, *p* = 0.012). Although addition of BV-2 CM in misfolded tau-expressing cells did not cause further increase of tau phosphorylation at Ser202, Thr231, and Ser396, pretreatment of SG-Tang could reverse abnormal tau hyperphosphorylation at Ser202 (76% vs. 107%, *p* = 0.022) and Thr231 (79% vs. 122%, *p* = 0.021) (Figure 6(g)). Our results demonstrate that SG-Tang could protect cells from cell death, increase neurite outgrowth, and reduce hyperphosphorylation of tau in inflamed misfolded tau-expressing ∆K280 tau_RD_-DsRed cells.

### 3.6. Identification of SG-Tang Targets by Human Apoptosis Antibody Array

**Figure 7:**
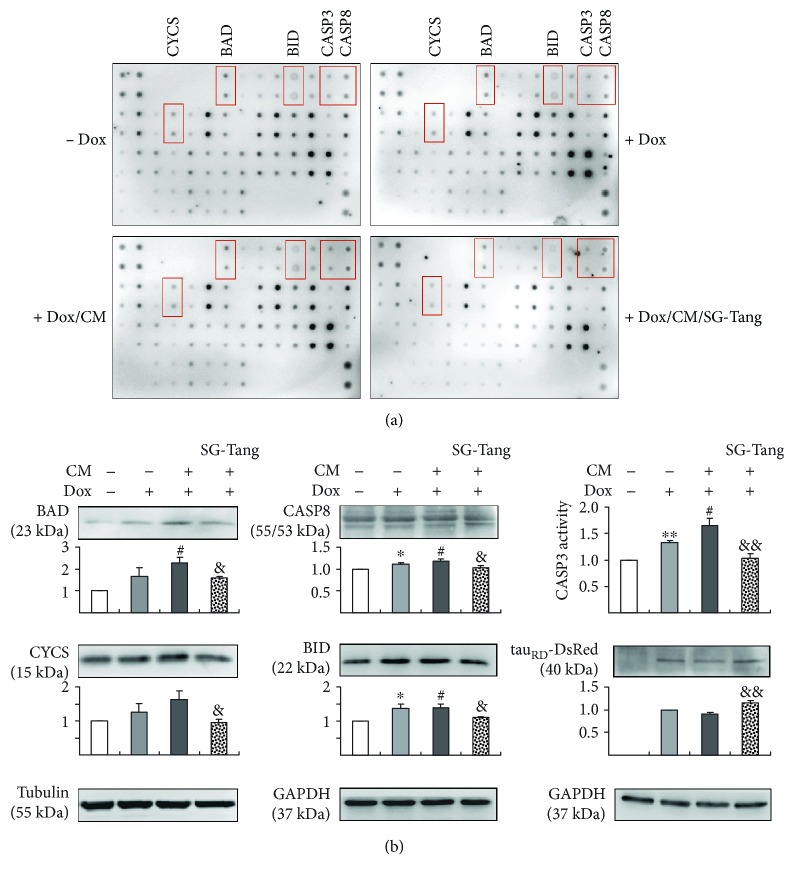
Apoptosis-related protein targets of SG-Tang in BV-2 conditioned medium-stimulated ∆K280 tau_RD_-DsRed SH-SY5Y cells. (a) Representative images of apoptosis antibody array of proteins collected from Figure 6(c). (b) Western blot analysis of BAD, CYCS, CASP8, BID, and tau_RD_-DsRed protein levels (normalized to tubulin or GAPDH internal control, *n* = 3) and caspase 3 activity assay from each sample. ^∗^ *p* < 0.05 and ^∗∗^ *p* < 0.01, **−** Dox vs. + Dox; ^#^*p* < 0.05, + Dox vs. + Dox/CM; ^&^*p* < 0.05 and ^&&^*p* < 0.01, + Dox/CM vs. + Dox/CM/SG-Tang-treated cells.

TNF-*α* has been long considered as an effecter of inflammation-induced cell death. It has been shown that TNF-*α* binds to receptor TNFR1 to permit the release of silencer of death domain (SODD) and the recruitment of intracellular death signaling inducing signaling complex (DISC) proteins, including TNFR-associated death domain protein (TRADD) and Fas-associated protein with death domain (FADD), which then activates caspase 8 leading to apoptosis [33]. Caspase 8 is also a key mediator of inflammation and processing of pro-IL-1*β* to IL-1*β* [34]. Since we have found SG-Tang decreased TNF-*α* and IL-1*β* in CM of BV-2, we proposed that SG-Tang may also act on the inflammation-induced cell death. To elucidate the molecular mechanisms underlying the rescue from inflammation-induced cell death by SG-Tang, proteins from uninduced (− Dox), induced (+ Dox), inflamed (+ Dox/CM), and SG-Tang-pretreated inflamed (+ Dox/CM/SG-Tang) ∆K280 tau_RD_-DsRed SH-SY5Y cells were examined by using human apoptosis array to evaluate expression levels of 43 apoptosis-related proteins (Figure 7(a)). Among these targets, expression of proapoptotic Bcl2-associated agonist of cell death (BAD), BH3-interacting domain death agonist (BID), caspase 3 (CASP3), caspase 8 (CASP8) and cytochrome c, and somatic (CYCS) were apparently reduced by SG-Tang treatment (Table 1). Western blot analysis of BAD, BID, CASP8, and CYCS expression changes and caspase 3 activity assay further confirmed that pretreatment of SG-Tang could significantly decrease these identified targets (BAD: from 228% to 157%, *p* = 0.023; BID: from 139% to 110%, *p* = 0.038; CASP8: from 118% to 104%, *p* = 0.024; CYCS: from 163% to 96%, *p* = 0.040; caspase 3 activity: from 165% to 103%, *p* = 0.005). Moreover, addition of SG-Tang improved ∆K280 tau_RD_-DsRed misfolding and enhanced soluble tau_RD_-DsRed protein level in inflamed ∆K280 tau_RD_-DsRed SH-SY5Y cells (from 90% to 115%, *p* = 0.004) (Figure 7(b)). Our results indicated that SG-Tang may protect inflamed ∆K280 tau_RD_-DsRed SH-SY5Y cells by inhibiting production of proapoptotic proteins.

## 4. Discussion

In this study, we demonstrated neuroprotection, antioxidative and anti-inflammatory effects of formulated CHM SG-Tang. Our results showed that SG-Tang displayed a greater antioxidative and antiaggregation effect than Bai-Shao and Gan-Cao and a stronger anti-inflammatory activity than Bai-Shao but similar to Gan-Cao (Figures 2 and 3). Moreover, SG-Tang showed neuroprotective effect of promoting neurite outgrowth probably by ameliorating tau misfolding and oxidative stress in our tauopathy model (Figures 3 and 4). The anti-inflammatory effects of SG-Tang were further demonstrated by using LPS-stimulated BV-2 microglia (Figure 5). Targets identified from human apoptosis protein array indicate SG-Tang may suppress the expression levels of proapoptotic proteins in inflamed *Δ*K280 tau_RD_-DsRed SH-SY5Y cells and thus elevate the cell viability (Figures 6 and 7).

In human tauopathy, substantial activated microglia are found in regions of phosphorylated tau accumulation [35]. In tau P301S transgenic mice, prominent glial activation precedes tangle formation and the pattern of activated glia correlates closely with the distribution and density of NFTs [36]. As neuroinflammation is linked to the progression of tauopathy, anti-inflammatory strategy may be effective at reducing tau-related pathology. Indeed, FK506 attenuates tau pathology and increased lifespan in tau P301S mouse model [36]. Treatment of 3xTg-AD mice with anti-inflammatory drug ibuprofen reduces tau phosphorylation and memory impairment [37]. Administration of potent anti-inflammatory minocycline reduces the development of disease-associated tau species in the htau mouse model [38] by reducing several inflammatory factors [39]. In the present study, we applied BV-2 conditioned medium to proaggregant ∆K280 tau_RD_-DsRed 293/SH-SY5Y cells to mimic neuroinflammation. The study results reveal that CHM formula SG-Tang displays neuroprotection by exerting anti-inflammatory and antiapoptotic activities.

Inflammation is a double-edged sword. Inflammatory response could lead to activation of immune system and elimination of pathogens thereby reducing further cell loss. Although inflammation might be protective and beneficial to cells, prolonged or dysregulated inflammatory process could also result in production of neurotoxic factors that exacerbate neurodegenerative pathology and cause cell death [12]. Thus, a potential strategy for treating tauopathies is to intervene in microglial activation and neuroinflammation. NSAID has been commonly used as treatment of inflammation and known to be neuroprotective [40]. The mechanism of NSAID has been shown to inhibit the synthesis or activity of inflammatory mediators such as prostaglandin and COX isoforms 1 and 2. Although NSAID could effectively suppress the inflammatory symptoms, these agents may also induce significant side effects such as increased risk of thrombotic cardiovascular and cerebrovascular events [41]. Therefore, more safely, anti-inflammatory drugs need to be explored and developed.

There is a growing interest in natural compounds/products with anti-inflammatory activities which have long been used for treating inflammation-related diseases. In this study, SG-Tang used was formulated with Bai-Shao (*P. lactiflora*) and Gan-Cao (*G. uralensis*) and analyzed by HPLC using two main active constituents, paeoniflorin and ammonium glycyrrhizinate (Figure 1). Both paeoniflorin and glycyrrhizinic acid were demonstrated to be able to cross the blood-brain barrier (BBB) in middle cerebral artery occlusion rats [42]. However, multiplicity of the components *in P. lactiflora and G. uralensis* contributes to the effects *of* antioxidation and anti-inflammation. In the root of *P. lactiflora*, a total of 40 components including 29 monoterpene glycosides, 8 galloyl glucoses, and 3 phenolic compounds were identified [43]. Among them, paeoniflorin, a monoterpene glycoside, is known to possess anti-inflammatory effect and has been applied to cerebral ischemic injury [44]. Paeoniflorin also exhibits neuroprotective effects via inhibiting neuroinflammation in APP/PS1 and in PS2 mutant mice [45, 46]. Paeoniflorin and the isomer albiflorin attenuated neuropathic pain by inhibiting the activation of p38 MAPK pathway in spinal microglia and subsequent upregulated IL-1*β* and TNF-*α* [47]. Benzoylpaeoniflorin, another paeoniflorin-related glycoside in *P. lactiflora* root, protected primary rat cortical cells against H_2_O_2_-induced oxidative stress [48]. In addition to monoterpene glycosides, gallic acid, a phenolic compound in *P. lactiflora* root, displayed antioxidative effect by scavenging free radicals, inhibiting lipid peroxidation, and protecting against oxidative DNA damage [49]. Paeonol, another phenolic compound in *P. lactiflora* root, exerted neuroprotective effect in the model of ischemia through reducing proinflammatory receptors/mediators [50].

The main bioactive components of *G. uralensis* are triterpene saponins and various types of flavonoids, including glycyrrhetinic acid, glycyrrhizic acid, liquiritigenin, isoliquiritigenin, liquiritin, and licochalcone A [51]. Glycyrrhizin and related compounds were found to show anti-inflammatory activity *in vitro* [52] and *in vivo* [53]. Although diammonium glycyrrhizinate rescues neurotoxicity in A*β*_1–42_-induced mice [54], its effect in tauopathy models is not known. Isoliquiritigenin, isoliquiritin, and liquiritigenin significantly suppressed iNOS, TNF-*α*, and IL-6 expression in IL-1*β*-treated rat hepatocytes [55]. Interestingly, the purified glycyrrhiza polysaccharides increased the pinocytic activity, the production of NO, IL-1, IL-6, and IL-12 in macrophages of mice [56]. Glycyrrhetinic acid, liquiritigenin, isoliquiritigenin, and liquiritin were also found to be all potent NRF2 inducers [57]. Moreover, Calzia et al. has shown that polyphenolic phytochemicals displayed a potent antioxidant action by modulating the ectopic F_0_F_1_-ATP synthase activity of the rod outer segments of the retina and prevented the induction of apoptosis [58]. Therefore, polyphenolic compounds from Bai-Shao and Gan-Cao may also exert antioxidative activities not only in but also outside of mitochondria. Given that multiple different compounds in both Bai-Shao and Gan-Cao are exerting effects on different pathways, the combination of Bai-Shao and Gan-Cao may thus have additive protection effects than each alone, which is supported by our study results.

The anti-inflammatory effect of Jakyakgamcho-tang, a formulated *P. lactiflora* and *G. uralensis* in Korea, has been shown by inhibiting the NF-*κ*B signaling pathway in keratinocytes [59]. Aberrant activation of NF-*κ*B signaling may lead to apoptosis and cell death [60]. We found that several proapoptotic proteins including BAD, BID, CASP3, CASP8, and CYCS were induced by misfolded tau expression and/or caused by LPS/IFN-*γ*-stimulated BV2 microglia. BAD protein is a proapoptotic member of the Bcl-2 gene family involved in initiating apoptosis [61]. BID is also a proapoptotic protein which plays a role as a sentinel for protease-mediated death signals [62]. Caspases are well-studied important mediators of apoptosis. CYCS is known to be released from mitochondria into cytosol to stimulate cell apoptosis [63]. Administration of SG-Tang decreased the production of these proapoptotic proteins, indicating that SG-Tang may target on inhibiting proapoptotic proteins to protect neuron cells from inflammatory damage.

Finally, pretreatment of SG-Tang reversed abnormal hyperphosphorylation at tau Ser202 and Thr231 in inflamed misfolded tau-expressing SH-SY5Y cells (Figure 6). Tau function is regulated by phosphorylation at specific sites, and tau phosphorylation plays both physiological and pathological roles in the cells. Tau Ser199/202 and Thr205 were found to be locally phosphorylated along the nascent axon during axonogenesis [64]. Phosphorylation of tau Thr231 inhibited tau to bind and stabilize microtubules [65]. Both Ser202 and Thr231 are hyperphosphorylated in degenerating AD brain [66]. Among kinases that regulate tau Ser202 and Thr231 phosphorylation, cyclic AMP-dependent protein kinase (PKA) and cyclin-dependent kinase 2 (CDC2) might be the potential targets of SG-Tang, and SG-Tang treatment may result in activity suppression of these two kinases [66, 67]. The exact mechanism for PKA or CDC2 regulation by SG-Tang remains to be determined in our future work.

## 5. Conclusions

Plant-derived natural medications have been used for centuries and becoming more popular because of their low side effects. Despite the fact that natural compounds are relatively safe, the complexity of natural products makes nutraceutical preparations difficult to be appropriately designed. In this study, we showed antioxidative and anti-inflammatory effects of SG-Tang as a potential agent for treatment or prevention of neuroinflammation-associated tauopathy. In future, studies of main active compounds paeoniflorin and ammonium glycyrrhizinate in SG-Tang, separately or in combination, in tauopathy cell model are warranted to provide a novel avenue for protection against tauopathy.

## Figures and Tables

**Table 1:** Proteins identified by human apoptosis antibody array.

Gene symbol	UniProt accession number	Protein	Fold change (+ Dox/CM vs. − Dox)	Fold change (+ Dox/CM/SG-T vs. + Dox/CM)
BAD	Q92934	Bcl2-associated agonist of cell death	1.16	0.63
BID	P55957	BH3-interacting domain death agonist	1.33	0.41
CASP3	P42574	Caspase 3	1.49	0.71
CASP8	Q14790	Caspase 8	1.34	0.62
CYCS	P99999	Cytochrome c, somatic	0.92	0.62

## Data Availability

The data used to support the findings of this study are available from the corresponding author upon request.

## Abbreviations

AD: Alzheimer's disease
BAD: Bcl2-associated agonist of cell death
BBB: Blood-brain barrier
BID: BH3-interacting domain death agonist
BSA: Bovine serum albumin
CASP3: Caspase 3
CASP8: Caspase 8
CDC2: Cyclin-dependent kinase 2
CHM: Chinese herbal medicine
CM: Conditioned medium
CNS: Central nervous system
COX: Cyclooxygenase
CYCS: Cytochrome c, somatic
DAPI: 4′-6-Diamidino-2-phenylindole
Dox: Doxycycline
DMEM: Dulbecco's modified Eagle's medium
DPPH: 1,1-Diphenyl-2-picrylhydrazyl
EC_50_: Half maximal effective concentration
ELISA: Enzyme-linked immunosorbent assay
FBS: Fetal bovine serum
HCA: High-content analysis
HPLC: High-performance liquid chromatography
Iba1: Induction of brown adipocytes 1
IC_50_: Half maximal inhibitory concentration
IFN: Interferon
IL: Interleukin
LDH: Lactate dehydrogenase
LPS: Lipopolysaccharide
MTT: 3-(4,5-Dimethylthiazol-2-yl)-2,5-diphenyltetrazolium bromide
NO: Nitric oxide
NSAID: Nonsteroidal anti-inflammatory drug
PKA: Cyclic AMP-dependent protein kinase
ROS: Reactive oxygen species
SG-Tang: Shaoyao Gancao Tang
tau_RD_: Tau repeat domain
TNF: Tumor necrosis factor.
